# Supplementary material for: Whole-genome screens reveal regulators of differentiation state and context-dependent migration in human neutrophils
Source: Nat Commun. 2023 Sep 18;14:5770. doi: 10.1038/s41467-023-41452-x (PMC10507112; doi:10.1038/s41467-023-41452-x)
Supplement: Supplementary file 1 — Supplementary Tables and Figures [file 41467_2023_41452_MOESM1_ESM.pdf]

<u>Supplementary Information</u>

Whole-genome screens reveal regulators of differentiation state and context-dependent migration in human neutrophils

Nathan M. Belliveau[1], Matthew J. Footer[1], Emel Akdogan[2], Aaron P. van Loon[1], Sean R. Collins[2], and Julie A. Theriot[1,*]

[1] Department of Biology and Howard Hughes Medical Institute, University of Washington, Seattle, WA 98195, USA

[2] Department of Microbiology and Molecular Genetics, University of California, Davis, Davis, CA 95616, USA

* Correspondence: jtheriot@uw.edu

Supplementary Tables

Supplementary Table 1: Differential expression of macrophage-relevant genes in FLCN and LAMTOR1 knockdown cell lines. Differential expression of genes in FLCN and LAMTOR1 knockdown line relative to control cells show a change in expression that is consistent with a shift toward macrophage differentiation. Log_2 fold-changes from RNA-seq data in dHL-60 cells are shown for genes identified as relevant to a macrophage cell type. Genes were also identified that are expected to show differential expression during differentiation toward either a neutrophil or macrophage cell type using RNA-seq data available from Ramirez et al. 2017, *Cell Systems* (PMID: 28365152).

**Commonly reported surface markers for macrophage cell type.** (i.e. identified in reference antibody panel cat# ab254013 from Abcam and a resource from Bio-Rad, https://www.bio-rad-antibodies.com/macrophage-m1-m2-tam-tcr-cd169-cd-markers-antibodies.html)		
Gene	Log2 fold change (day5+DMSO, sgFLCN vs sgControl)	Log2 fold change (day5+DMSO, sgLAMTOR1 vs sgControl)
CD163	3.62	3.52
CCR5	2.93	4.04
CD14	-0.67	-1.41
CD16 (FCGR3A)	not present in data	-2.63
CD64 (FCGR1)	0.86	0.42
CD71 (TFRC)	2.17	2.0
**Genes identified in RNA-seq data from Ramirez et al. 2017, *Cell Systems* (PMID: 28365152) that show increased expression in macrophages, decreased expression in neutrophils.**		
Gene	Log2 fold change (day5+DMSO, sgFLCN vs sgControl)	Log2 fold change (day5+DMSO, sgLAMTOR1 vs sgControl)
CSF1	1.1	1.62
CD52	3.23	3.77
EMP1	2.4	3.62
CD9	1.36	3.16

FCRLA	4.82	5.52
FCRLB	0.78	1.52
ACTN1	0.30	0.45
MYO1C	0.47	0.47
**Genes identified in RNA-seq data from Ramirez et al. 2017, *Cell Systems* (PMID: 28365152) that show decreased expression in macrophages, increased expression in neutrophils.**		
S100A8	-1.18	-2.40
PKN1	-0.58	-0.77
MYADM	-0.34	not present in data
SLC9A3R1	-0.60	-0.86
TESC	0.24	-0.36
CSF3R	-0.72	-1.47
CD4	-0.23	-0.53
FGR	-0.25	-0.20
LRG1	-0.67	-0.82
LSP1	0.15	0.15

Supplementary Figures

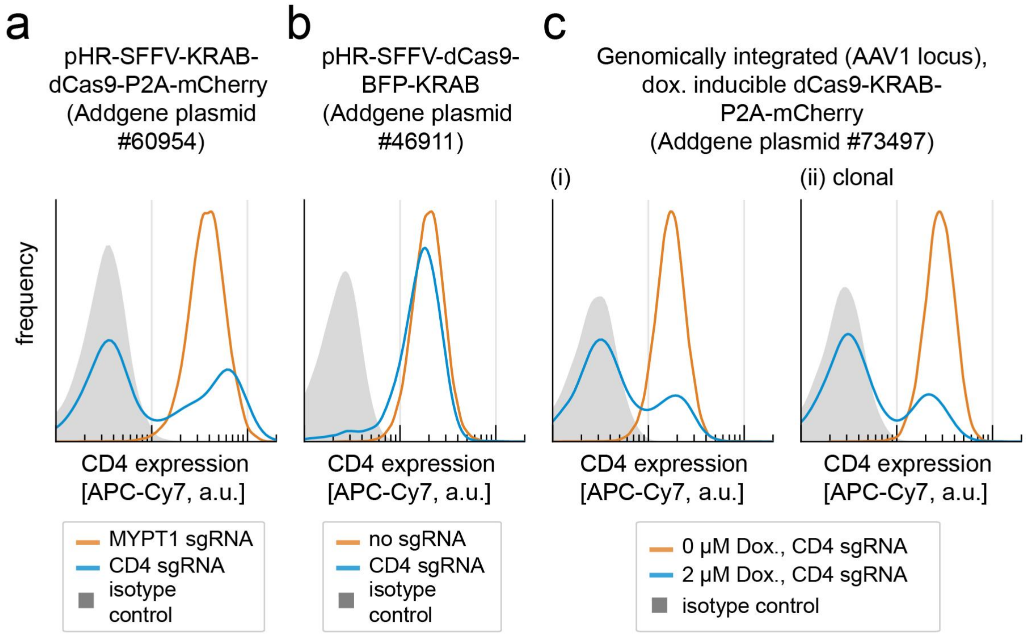

Supplementary Figure 1. **Comparison of gene knockdown across different dCas9-KRAB constructs. a** Flow cytometry measurements of cell surface CD4 protein expression using three other dCas9-KRAB constructs are shown. Normal expression of CD4 in uHL-60 cells is shown in orange, while cells carrying a sgRNA targeting CD4 are shown in blue. Background autofluorescence and non-specific fluorescence were determined using an isotype antibody control (gray, shaded). **b** dCas9-KRAB-P2A-mCherry driven by the SFFV promoter (Addgene plasmid #60954). This construct was genomically integrated by lentiviral transduction following the same method described for the construct of the main text. **c** dCas9-BFP-KRAB (Addgene plasmid #46911). This construct was genomically integrated by lentiviral transduction following the same method described for the construct of the main text. **d** dCas9-KRAB-P2A-mCherry driven by a doxycycline Inducible promoter (Addgene plasmid #73497). This was genomically integrated into the AAVS1 'safe harbor' locus using co-electroporation with ribonucleoprotein complex of (Cas9 protein and a sgRNA targeting AAVS1) as described in Mandegar et al 2016 (Cell Stem Cell 7;18(4): 541-53; PMID:26971820). The resulting polyclonal cell line (i) or clonal cell line (ii), generated by single-cell sorting, were tested for knockdown activity.

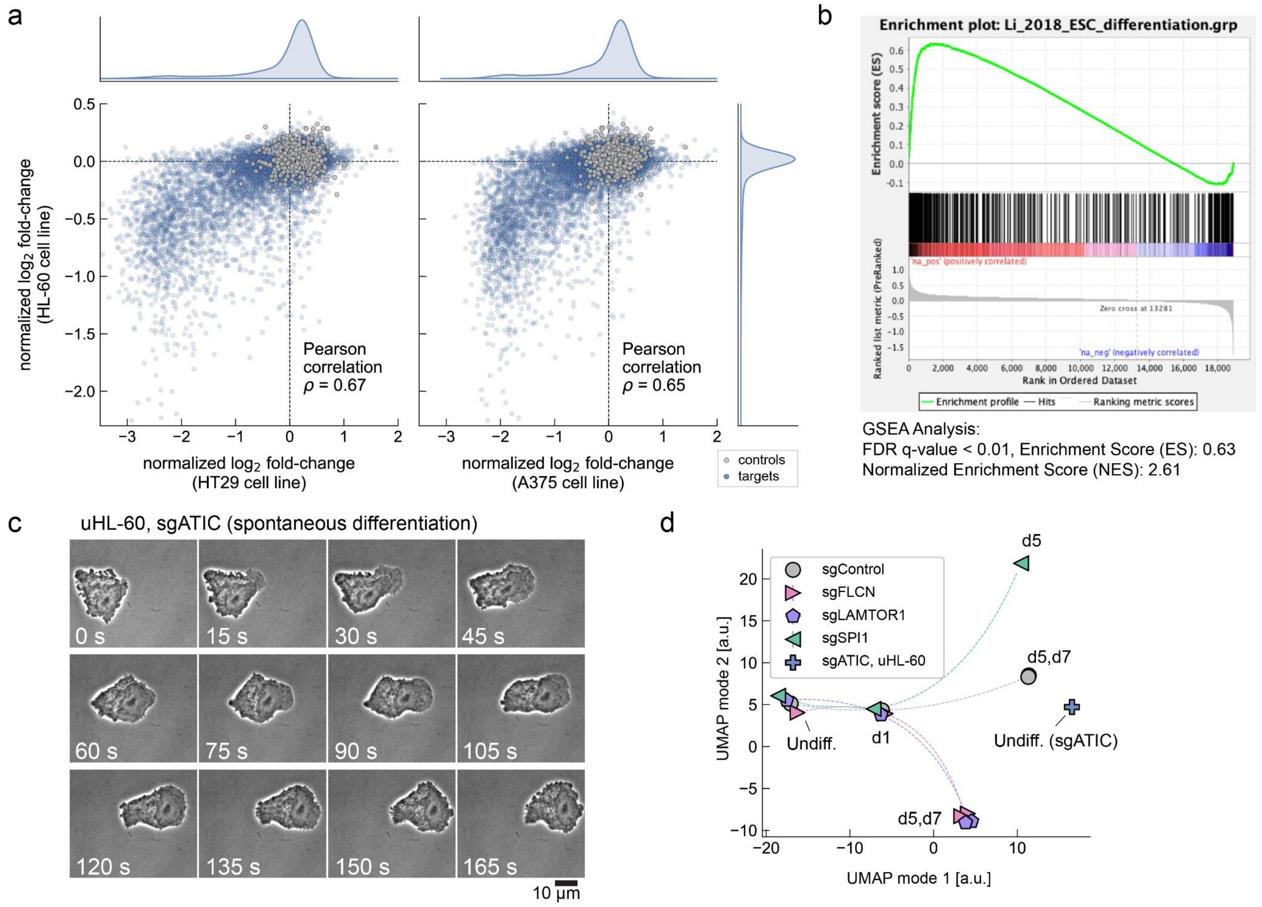

Supplementary Figure 2. **Additional analysis of cell proliferation and differentiation screens. a** Scatter plots show the normalized $\log_2$ fold-changes measured by comparing the change in sgRNA abundances following six days of growth (HL-60 cell line), plotted against the similar data from Sanson et al. 2018 (HT29, human colorectal adenocarcinoma cell line; A375, human melanoma cell line). Note that the normalized $\log_2$ fold-changes from Sanson et al. 2018 compares sgRNA abundance following 21 days of proliferation, compared to the initial distribution of sgRNAs in the plasmid library preparation. **b** Gene set enrichment analysis (GSEA) of differentiation data against the genes that were identified in a CRISPR knockout screen for exit from pluripotency in mouse embryonic stem cells (Li et al., 2018; PMID: 29996108). The figure shows an enrichment plot from the GSEA analysis software (Subramanian, Tamayo, et al., 2005; PMID: 16199517) showing a positive enrichment for genes identified in the Li et al. study. **c** Time lapse of phase microscopy images of an uHL-60 cell with sgRNA targeting ATIC for gene knockdown. The cell was placed under an agarose overlay in order to confine the cell and prevent movement/detachment due to fluid flow. **d** The plot shows the addition of uHL-60 cells with sgRNA targeting ATIC to the UMAP dimensionality reduction analysis from the main text (‘+’ symbol), which places them more in line with differentiated dHL-60 cells. Individual data points represent an average across 6 RNA-seq replicate samples.

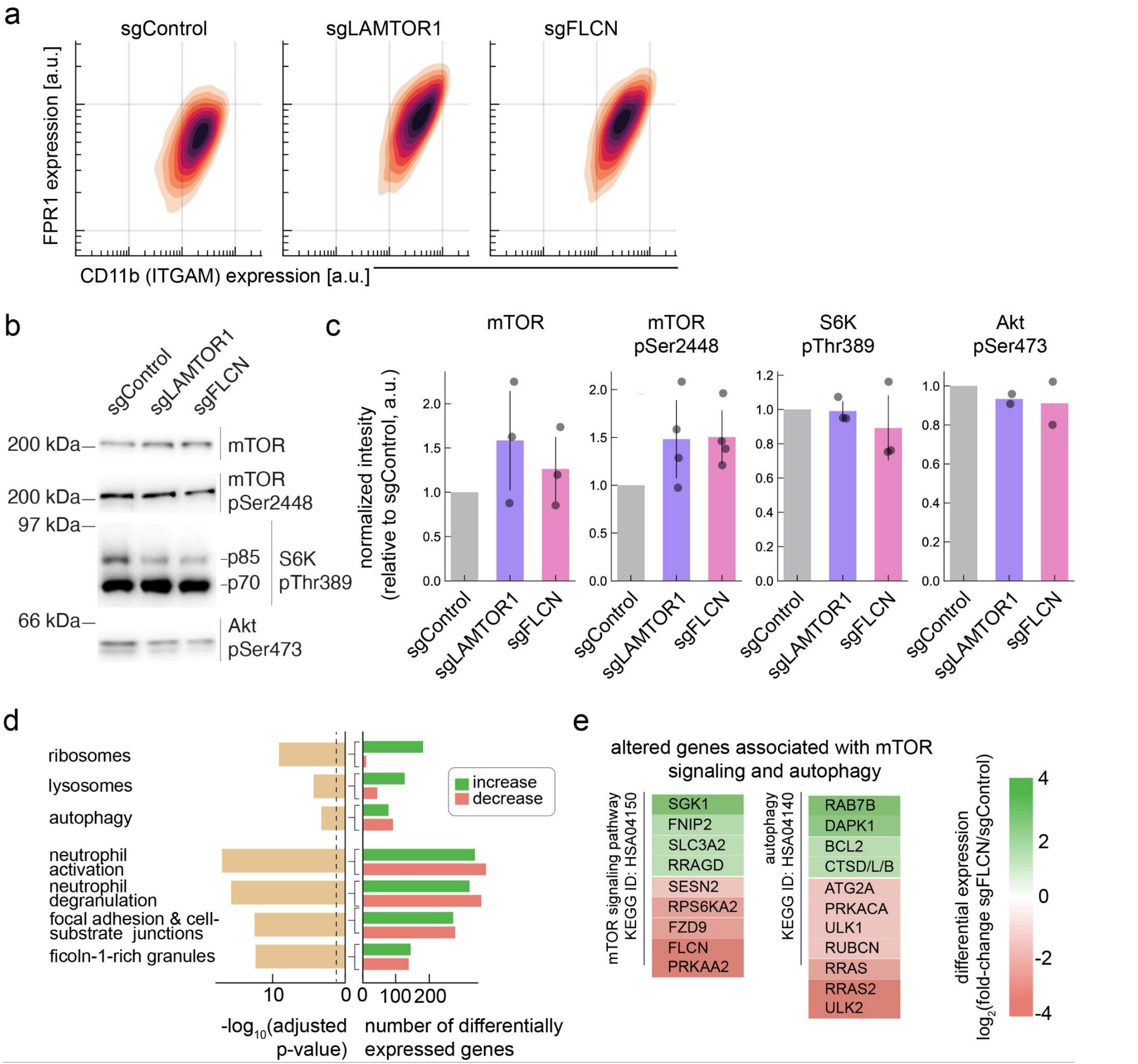

Supplementary Figure 3. **Additional analysis of FLCN and LAMTOR1 knockdown lines.** **a** Flow cytometry immunofluorescence measurements of CD11b (ITGAM) and fMLP receptor (FPR1) cell surface expression in dHL-60 cells containing a control sgRNA, LAMTOR sgRNA, and FLCN sgRNA. **b** Western blots assaying mTORC1/2 activity in differentiated neutrophils (7-day post differentiation) in knockdown lines targeting LAMTOR1, FLCN, and sgRNA control. S6K is a target of mTORC1, while Akt is a target of mTORC2. p85 and p70 correspond to two protein isoforms of S6K. **c** Quantitation of multiple Western blots as in part (**b**). Western blot band intensities were normalized to total protein loading, estimated from an Alexa Fluor 790 GAPDH antibody. Relative normalized intensity values are compared to the sgControl cell line. Bar plot shows the mean +/- SD for, while individual points represent the individual replicate blots (mTOR, n = 3; mTOR pSer2448, n = 4; S6K pThr389, n = 3; Akt pSer473, n = 2; No estimate for standard deviation is provided for Akt pSer47). Quantitation of S6K protein included band intensities for both p85 and p70 isoforms. Analysis was performed using Bio-Rad's Image Lab software (v. 6.1). **d** A subset of the pathways identified based on their differential expression between FLCN knockdown cells and control sgRNA (comparison

made between day 5 differentiated cells. Gene set p-values estimate the statistical significance of gene set enrichment, calculated using one-sided permutation tests and adjusted for multiple comparisons using the Benjamini–Hochberg procedure. **e** Subset of the most differentially expressed genes that were identified in the comparison between FLCN knockdown cells and control sgRNA that relate to mTOR signaling and autophagy. For (**d**) and (**e**), differentially expressed genes were identified using a one-sided Wald test to compare gene expression in the FLCN knockdown and control cell lines. P-values were adjusted for multiple comparisons using the Benjamini–Hochberg procedure and considered significant if less than 0.05.

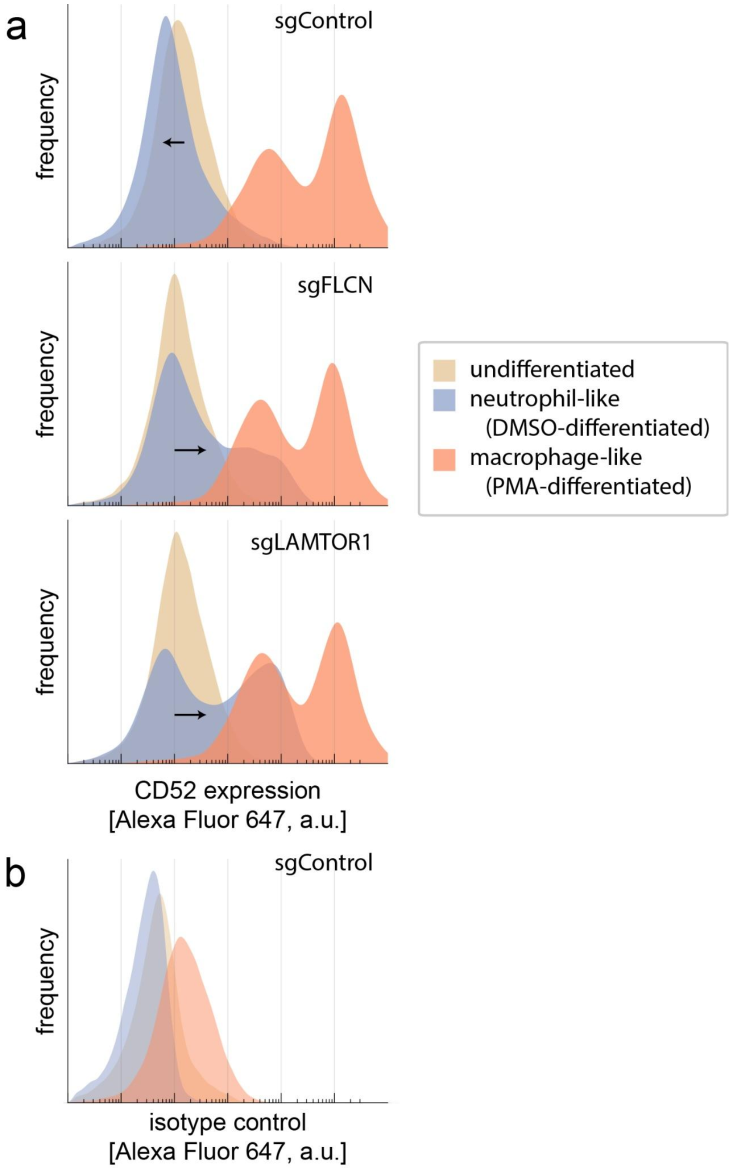

Supplementary Figure 4. **Analysis of CD52 protein expression during differentiation of uHL-60 cells into dHL-60 neutrophil-like and macrophage-like cells (complement to RNA-seq expression changes in Supplementary Table 1). a** Surface expression of CD52 was measured by immunofluorescence flow cytometry (see *Methods* section of main text for additional details). Neutrophil-like cells were prepared by inducing differentiation with DMSO as described in the main text. Macrophage-like cells were generated by incubating cells with phorbol 12-myristate 13-acetate (PMA, MilliporeSigma, #P1585) at a concentration of 50 ng/mL for 5 days. For both differentiation conditions, media was replaced with fresh media, including the differentiation reagent, at day 3 following the initiation of differentiation. Black arrows qualitatively indicate the sign shift in expression following DMSO mediated differentiation. **b** Immunofluorescence flow cytometry of undifferentiated uHL-60 cells, dHL-60 neutrophil-like cells, and dHL-60 macrophage-like cells using an isotype control. This plot highlights that some of the observed change in signal

intensity is likely due to differences in autofluorescence or antibody binding across the different cell types.

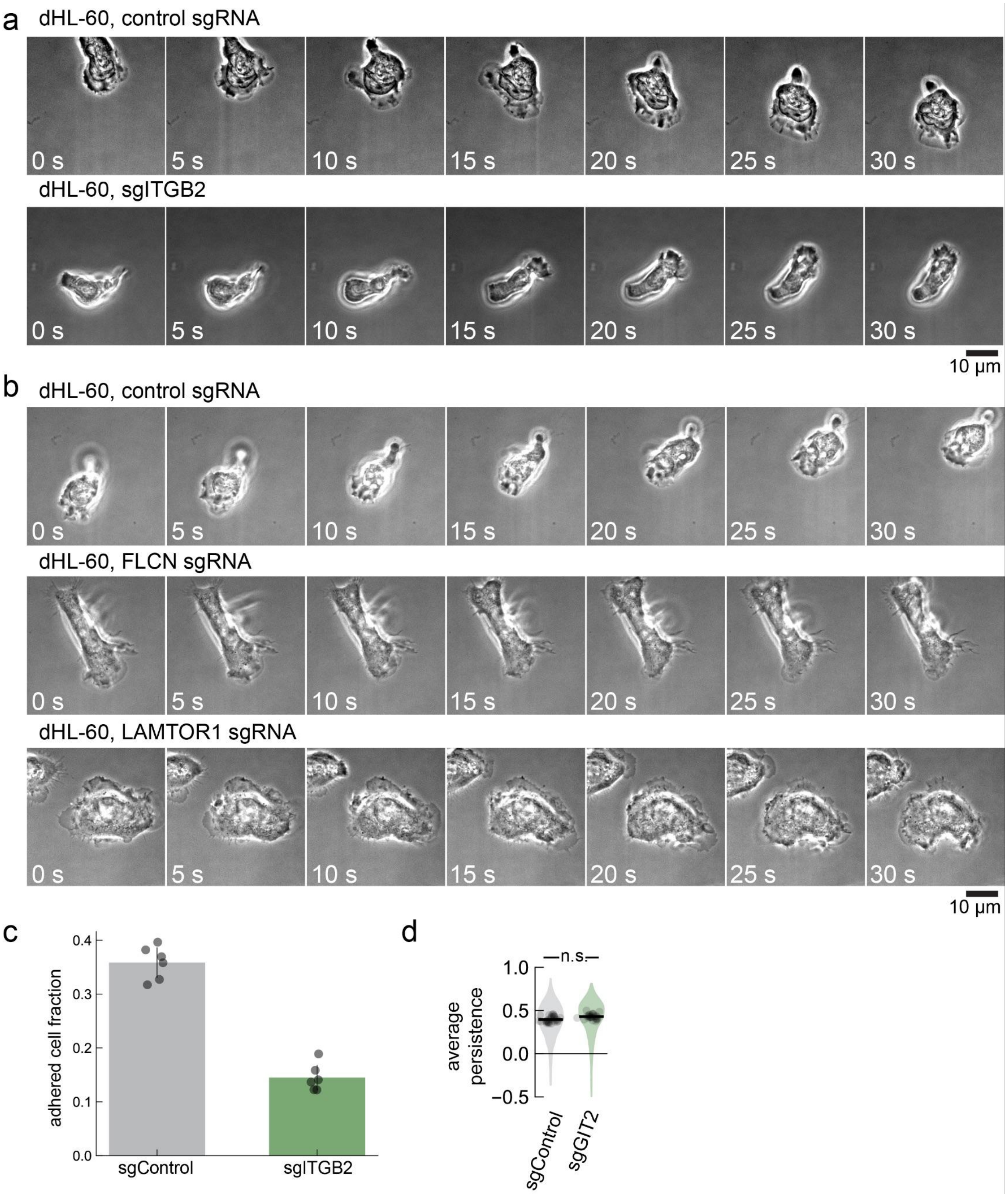

Supplementary Figure 5. **Additional characterization of cell migration in sgRNA knockdown cell lines. a** Time lapse of phase microscopy images for dHL-60 cells expressing either a control sgRNA (top) or sgRNA targeting ITGB2 for gene knockdown (bottom). Coverslips were coated with fibronectin (see Methods). The phase-bright feature of the ITGB2 knockdown line is because the cell is detached and rounded up, floating above the

coverslip. **b** Time lapse of phase microscopy images for dHL-60 cells expressing either a control sgRNA (top), sgRNA targeting FLCN (middle row), or sgRNA targeting LAMTOR1 (bottom). Coverslips were coated with fibronectin (see Methods in the main text). **c** Fraction of adhered cells following incubation of cells for 1 hr in a tissue culture treated multiwell plate. Error bars indicate mean values +/- SD across 6 individual wells where independent adhesion assays were performed. Data points correspond to measurements from individual wells. **d** Characterization of cell migration speed following knockdown of GIT2. Persistence was inferred from the cell velocity data as described by Metzner et al. (see Methods in the main text). Individual data points represent mean values for cells across individual fields of view, with the shaded regions showing the distribution of all measurements (n.s. $p > 0.05$). Measurements represent experiments performed over 3 days, acquired across 29 fields of view.

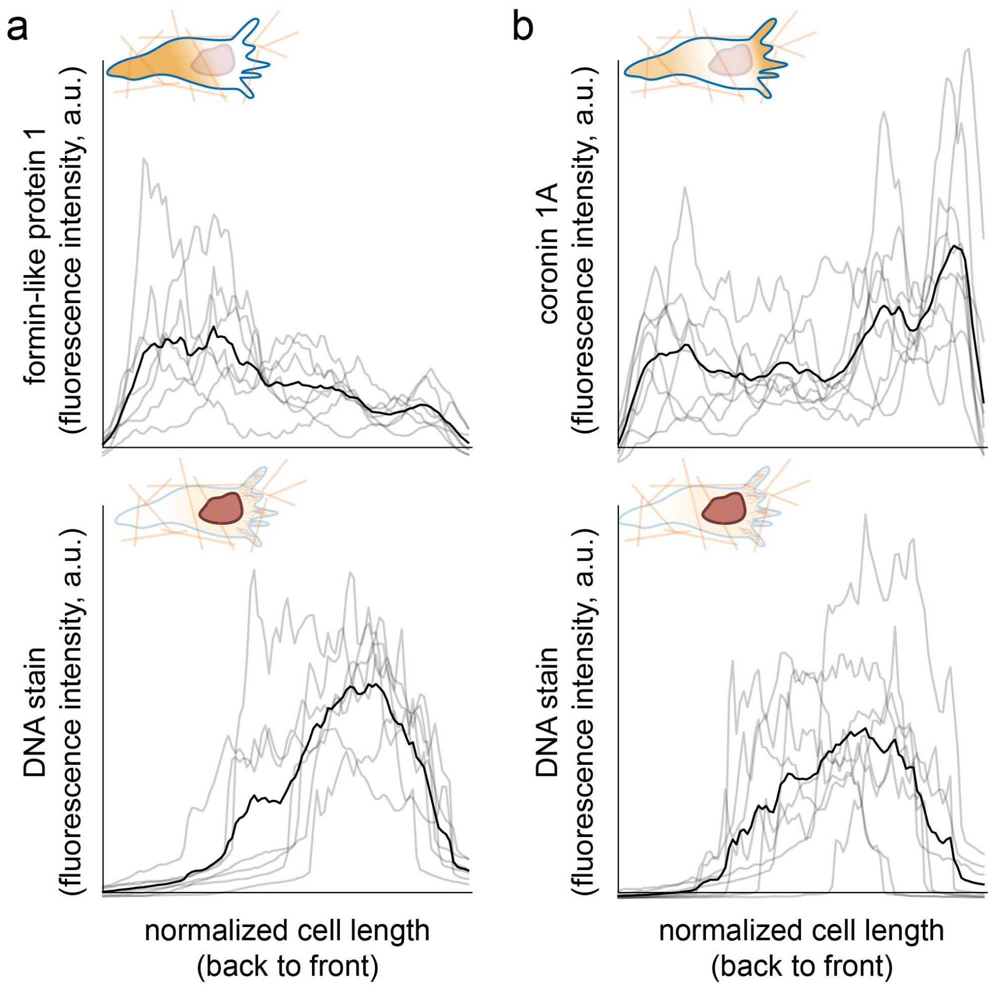

Supplementary Figure 6. **Additional characterization of immunofluorescence images for formin-like 1 and coronin 1A during migration in collagen.** **a** Histograms of immunofluorescence images for formin-like protein 1 (top) and DAPI DNA stain (bottom) across 7 cells. The dark line represents an averaged histogram across all cell data. Schematic insets identify the localization of fluorescent signal intensity. **b** Fluorescent histograms of immunofluorescence images for coronin 1a (top) and DAPI DNA stain (bottom) across 8 cells. The dark line represents an averaged histogram across all cell data. Schematic insets identify the localization of fluorescent signal intensity.

For (**a**) and (**b**), Image data represent cells that were clearly migrating based on their polarization and generally more intense localization of F-actin at the cell front. In order to generate histograms, images were rotated to orient the cell horizontally, approximately as shown in the schematic illustration.

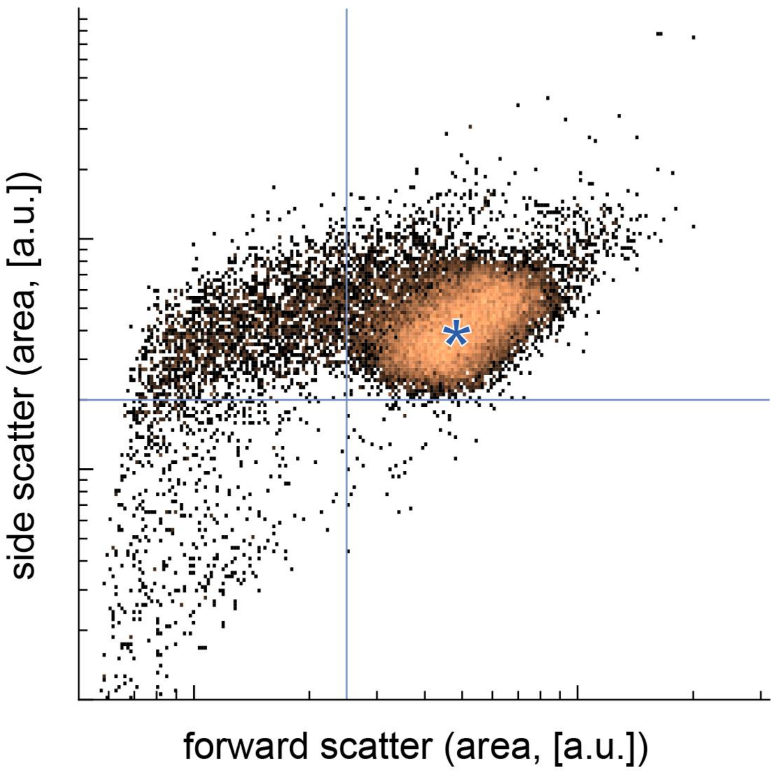

Supplementary Figure 7. **Illustration of gating for flow cytometry experiments.** An example plot of the forward scatter area measurement versus side scatter area measurement for uHL-60 cells (uHL-60 dCas9-KRAB without any sgRNA, used for Fig. 1a of the main text).The blue lines indicate the gating used, where only cells in the upper right quadrant containing (*) were used. Only this cell population was used for the generation of flow cytometry histograms.
