# Supplementary material for: Whole-genome screens reveal regulators of differentiation state and context-dependent migration in human neutrophils
Source: Nat Commun. 2023 Sep 18;14:5770. doi: 10.1038/s41467-023-41452-x (PMC10507112; doi:10.1038/s41467-023-41452-x)
Supplement: Supplementary file 3 — Description of Additional Supplementary files [file 41467_2023_41452_MOESM3_ESM.pdf]

Description of additional supplementary files

Supplementary Data 1-2
The .csv files contains the log2 fold-changes in sgRNA abundance for the screens of proliferation (Supplementary Data 1), differentiation (Supplementary Data 2). The sign for each gene indicates whether knockdown led to an enrichment of the associated sgRNA (positive) or depletion (negative).

Supplementary Data 3
The .csv file contains log2 fold-changes comparing the average differential expression between a dHL-60 FLCN knockdown cell line and dHL-60 control sgRNA cell line (six replicates each; 5 days following induction of differentiation). The file also includes the associated DESeq2 normalized RNA-seq expression data, gene identifiers and gene descriptors.

Supplementary Data 4
The .csv file contains log2 fold-changes comparing the average differential expression between a dHL-60 LAMTOR1 knockdown cell line and dHL-60 control sgRNA cell line (six replicates each; 5 days following induction of differentiation). The file also includes the associated DESeq2 normalized RNA-seq expression data, gene identifiers and gene descriptors.

Supplementary Data 5-7
The .csv files contains the log2 fold-changes in sgRNA abundance for the screens of chemokinesis (Supplementary Data 4), chemotaxis (Supplementary Data 5), and 3D amoeboid cell migration (Supplementary Data 7). The sign for each gene indicates whether knockdown led to an enrichment of the associated sgRNA (positive) or depletion (negative).

Supplementary Movie 1
The .avi file contains phase video microscopy associated with the snapshots of Fig. 5b and Figure S5a. Scale bar (10 µm) and acquisition time are identified in the video and apply to both videos, which show migration of dHL-60 cells with a control sgRNA or sgRNA targeting ITGB2 for gene knockdown.

Supplementary Movie 2
The .avi file contains phase video microscopy associated with the snapshots of Fig. 5c and Fig. S5b. Scale bar (10 µm) and acquisition time are identified in the video and apply to all three videos, which show migration of dHL-60 cells with a control sgRNA, sgRNA targeting FLCN, and sgRNA targeting LAMTOR1.
